# Locally implemented prevention programs may reverse weight trajectories in half of children with overweight/obesity amid low child-staff ratios: results from a quasi-experimental study in France

**DOI:** 10.1186/s12889-020-09080-y

**Published:** 2020-06-15

**Authors:** Aymery Constant, Gaëlle Boulic, Agnes Lommez, Raphaëlle Chaillou, Bernard Guy-Grand, Sandrine Raffin

**Affiliations:** 1 INRAE, INSERM, Univ Rennes, CHU Rennes, Nutrition Metabolisms and Cancer, NuMeCan, Rennes, France; 2 EHESP School of Public Health, Rennes, France; 3 Vivons en forme Association, 474 Saint-André-Lez-Lille, France; 4 Paris University Hospital, Paris, France; 5 LinkUp company, Levallois, France

**Keywords:** Obesity, Prevention, Child health, Deprivation, Effectiveness, Quasi-experimental study, Schools, Process and outcomes analysis

## Abstract

**Background:**

The aims of the present study were to assess changes in weight status between the first and last year of primary education among children with overweight/obesity in response to locally implemented school-based prevention programs, and to assess the influence of process indicators, expressed as child-staff ratios (CSRs), on these changes.

**Methods:**

To meet the study objectives, a quasi-experimental design was used. Four municipalities that systematically monitored the weight status of schoolchildren and participated in the “Vivons en Forme” program agreed to provide the data available in their school medical service records. The local implementers involved in training sessions were mainly municipal staff in charge of serving midday school meals, which is compulsory in France, and those in charge of designing and facilitating creative, interactive activities at school between and after classes. CSRs were determined by occupation (school catering service/facilitator of extracurricular activities) and training session (healthy eating/physical activity) in each municipality program, and classified as low (1–5 children per adult) or moderate.

**Results:**

During the 4 years of primary education, weight status improved in half of the children with overweight/obesity, and worsened in 6.6% of children with overweight/normal weight. In children who remained overweight, the BMI z-score diminished over time. Estimates of the positive 4-year weight changes were related to low CSRs in locally implemented variations of the program. Estimates increased with age and were significantly higher in low-to-moderate CSR multicomponent interventions than moderate CSR single-component intervention (reference). The moderate CSR multicomponent intervention had a similar effect as the reference. The estimated negative weight change decreased with age.

**Conclusions:**

Our findings suggest that training ancillary school staff in experiential-focused interventions for healthy eating and physical activity in locally implemented school-based programs contributed positively to reducing childhood obesity during the four years of primary education without interfering with educational activities. The results also provide preliminary evidence that low CSRs could be pivotal for optimal outcomes, especially in deprived areas.

## Background

The rates of obesity and overweight are still increasing worldwide [1], and remain unchanged in France, affecting nearly 1 in 5 children, with the highest prevalence in deprived populations [2]. Obesity in childhood is difficult to reverse [3] and often persists into adulthood, causing many health problems [4]. According to systematic reviews, school-based prevention programs may be effective in promoting healthy behaviors [5–7]. These programs typically include educational, environmental, and social activities designed to improve dietary habits and reduce sedentary time in schoolchildren [8, 9]. However, even sound, evidenced–based interventions yield modest effects on adiposity measures [10, 11].

Insights from implementation science may partly explain these mitigated outcomes. How authorities bring prevention programs into the school communities may dramatically influence local dynamics. In France, the Education, Health, and Territory (EST) program included the core principles of health-promoting schools, such as staff training and support to develop school health policies focusing on the school environment and adaptation to a local context, community involvement, and the development of health-related knowledge, skills, and competencies [12], but decisions and approvals came from higher authorities, and this “top-down” approach created a reluctance to participate locally [13]. In addition, health education programs require teachers to acquire additional competencies [14] and to include health education in their curriculum [15]. However, teachers’ work is already described as increasing in complexity and intensity because of societal changes, reformed and increased work tasks, and multitasking [16]. Urgent unforeseen priorities, competing responsibilities, and high workload may also constitute barriers to successful implementation [17], especially when training and activities seem complex or theoretical [13].

The success of school-based prevention initiatives involves balancing evidence-based interventions with the flexibility to permit local educational communities to target their specific needs [18]. Fostering commitment entails giving local implementers the freedom to select the intervention components that best meet their needs and providing training to persons interacting with children [19], including teachers and ancillary staff in charge of non-teaching duties at school [13], such as catering and extracurricular activities. Following these core principles, some associations provide organizational backbone support [20] to local communities in charge of the education and care of children. This alternative approach to downstream interventions may induce variation between locally implemented programs, warranting a thorough examination of the relationship between process and outcome indicators through a quasi-experimental study design [21].

The aims of the present study were to assess changes in weight status among schoolchildren between the first and last year of primary education in response to locally implemented school-based prevention programs, and to assess the influence of process indicators, expressed as child-staff ratios (CSRs) by occupation/component, on these changes.

## Methods

### Intervention

The *Vivons en Forme* (VIF; “live healthy”) organization is a community-based prevention program aimed at promoting healthier lifestyles among children and their families, and involves municipal service in charge of child education and care under the supervision of a local coordinator. VIF is a continuation of the obesity prevention scheme previously known as Epode [22] in which local actors distributed toolkits fostering educational messages. However, providing information is modestly effective in changing behavior [23], and the non-governmental organization acting as a backbone structure changed its process in 2010, following four new pathways in order to improve program efficiency. First, the name of the program was changed for greater acceptance by the local stakeholders, including families and children, removing the mention of obesity in the name of the interventions. Second, a full social marketing approach was included for each yearly implemented theme [24]. Third, toolkit materials were pilot-tested in living labs to collect input from users and stakeholders before application in real-life settings and the participating cities [25]. Lastly, the implementation process was centered around local stakeholders, including school staff, as well as participation and empowerment [26]. The principle aim was to foster self-efficacy and a long-lasting effect in local school staff newly involved in the field of prevention and health promotion. Local coordinators have the freedom to select the components on which they want to focus their interventions and can request additional interventions during the course of the program. The basic underlying principle of this “choose-and-pick” approach was to foster staff involvement and sustainably change their interactions with children and parents. Each participating municipality applies for a minimal 5-year period, and their representatives have to regularly attend regional coordination meetings to receive up-to-date information on training sessions and tool upgrades.

### Study design and participant selection

**Fig. 1:**
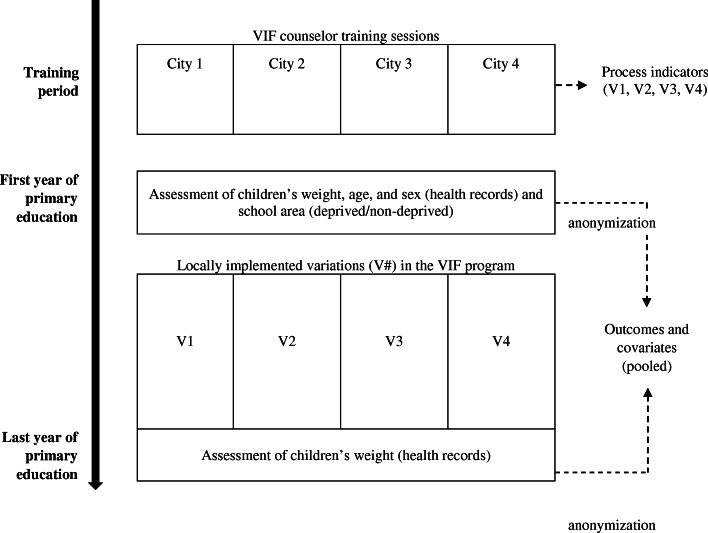
Quasi experimental study design

A quasi-experimental design was used was to meet the study objectives (Fig. 1). Four municipalities that systematically monitored the children’s weight status in the primary schools and were participating in the VIF program agreed to provide the data available in their school health records. In these municipalities, VIF counselors (a nutritionist, a sociologist, and the leading coordinator of the program) organized training sessions for the municipal staff in charge of school catering and extracurricular activities (ECAs) in primary schools. Training sessions and toolkits integrated roadmaps for conducting interactive activities with the children and to reinforce child-staff interactions via concrete experiences (Table 1). Brochures highlighting the beneficial effect of healthy eating (HE) and physical activity (PA) for children were systematically provided to parents [26]. They included tips on how to help kids stay hydrated by drinking water, on breakfast preparation, food breaks (including fruit), avoiding snacking between meals, on treats and smart portion sizes, and how to easily cook healthy meals at low cost.

**Table 1:** Description of training and tools provided to local school staff between the first (2011) and last year (2015) of primary education by occupation and thematic component

Occupation	Extracurricular activities		School canteens
Component	Physical activity	Healthy eating	Healthy eating
Training session	10 activities designed to involve kids in active gaming at school	Focus on 5 activities: • Food rhythms and snacking control • Morning snacking management • Fostering breakfast consumption • Healthy snacks, treat portions, and eating more fruit • Drink water everyday	• 48 tips for canteen cooks to cook tasteful vegetables For canteen staff and staff in charge of extracurricular activities during meal time and leisure centers • Portion size and indulging products • Eco-gestures (no waste) • Improving lunch-time break: time to experience more balanced eating habits
Tools	• Training booklet and various tools to implement active games	• For the staff: roadmaps to conduct interactive sessions with the children and brochure focusing on relevant food rhythms for children • For staff and children: posters reminding to avoid snacking between meals and to eat healthy food on break • For parents: dedicated booklets on how to manage treats and on drinking water	• For canteen staff: a training booklet to set up workshops, portion posters to remind them of the guidelines discovered during the training • For canteen cooks: a booklet with tips to cook vegetables and a brochure to answer their most common questions • For children: a charter of good conduct to experience pleasant canteen meals (noise, respect of others) • For parents: booklet with indication of portion sizes

### Measures

#### Weight status

School nurses received training and standardized weighing scales to measure body mass index (BMI) in children using a BMI chart established by the International Obesity Task Force, which allows classification of children into weight categories [27] (i.e., underweight, normal weight, overweight, and obesity). They assessed the weight status of children during the first year of primary education at the school premises several weeks before the launch of each program in 2011. Children wore light clothing and no shoes during the weighing sessions. In addition, BMI Z-scores were determined using BMI-for-age reference standards [28] in order to account for the severity of overweight/obesity. A second weight assessment was performed among the same children during the last year of their primary education in 2015.

#### Socio-demographics

Weight status was matched for sex and age at inclusion, and whether children were schooled in a zone of priority education (*zone d’education prioritaire*, ZEP) was indicated. ZEP refers to schools in deprived, usually urban, settings that are earmarked for special state support. The decision to categorize a school as a ZEP was left to the administrative authorities, who can release additional funding to finance special needs education. Households and individuals of lower socio-economic status (unemployed, single mothers) are overrepresented in ZEPs compared to other city areas.

#### Process indicators

The number and occupation (school catering or ECAs) of persons who attended training sessions between 2011 and 2015 were systematically recorded by thematic component (i.e., HE and/or PA).

### Data blinding and confidentiality

Each municipality provided data collected in the schools under their supervision without identifying a particular school or area. A study number was attributed to each municipality (City #) and each child in the database to ensure confidentiality. The final database was completed in 2016, but anonymized data were transmitted to researchers in charge of statistical analyses in 2018 due to the administrative authorization procedure in each participating city.

### Statistical analysis

Process indicators were expressed as number and occupation of school ancillary staff attending training sessions by thematic component in each municipality, and then converted into CSRs, the number of children to each trained staff member, for each occupation and thematic session. Because an average ratio of 8 children per adult was found in early childhood education and care settings [29]. The CSR was classified as “low” if between 1 and 5 children per adult, and “moderate” otherwise.

Categorical data were expressed as numbers and percentages and compared using the chi-squared test, or the McNemar test. Numerical data were expressed as means and standard deviations (SDs) and compared by one-way analysis of variance or the non-parametric Wilcoxon comparison test. Outcome indicators were 4-year changes in weight status, which were considered “positive” if obesity changed to overweight/normal weight or if overweight changed to normal weight, and “negative” if normal weight changed to overweight/obesity or if overweight changed to obesity. To investigate the influence of process indicators on weight changes, we entered each CSR (low, moderate) as a covariate in a logistic regression using positive 4-year weight change as the binary outcome (yes/no), with and without adjustments for age at inclusion, gender, and school area (deprived/non-deprived). To investigate the effect of combined CSRs, the four variations of the VIF program locally implemented in the participating municipalities were entered in a regression model as a categorical covariate, as collinearity precluded the use of multiple regression with interaction terms. The same statistical procedure was carried out with negative 4-year weight change as a binary outcome in children characterized as overweight/normal weight at inclusion. Estimates were expressed as odds ratios (ORs) with 95% confidence intervals (CIs). Statistical analyses were performed using the SPSS statistical package, version 20 (SPSS, Chicago, Illinois, United States).

## Results

Of the 900 first-grade children schooled in the four participating municipalities, 23 (2.7%) were not enrolled based on parental request, whereas 50 (5.6%) were lost to follow-up and excluded from further analyses. Comparisons revealed that those schooled in deprived areas were less frequently lost to follow-up (3.9%) than others (8.9%; *p* = 0.007). The 827 children included in the analyses (Table 2) were gender-balanced, with an average age of 6.4 years (SD 0.79), and 59.3% were schooled in deprived areas during the first year of primary education. The mean age was significantly higher in City 1 than the other municipalities (*p* < 0.001), whereas children schooled in deprived areas were significantly overrepresented in City 4 (77.4%) and City 1 (71.6%) compared to City 3 (45.3%) and City 2 (0%; p < 0.001). The distributions of gender and weight status were similar between municipalities.

**Table 2:** Characteristics of the schoolchildren in the first year of primary education (*N* = 827)

		Municipality				
	All	City 1	City 2	City 3	City 4	
	*N* = 827	*N* = 236	*N* = 137	*N* = 95	*N* = 359	*p*-value^a^
Sex						
Girls	404 (48.9)	115 (48.7)	70 (51.1)	47 (49.5)	172 (47.9)	0.936
Boys	423 (51.1)	121 (51.3)	67 (48.9)	48 (50.5)	187 (52.1)	
Mean age, years (SD)	6.38 (0.76)	6.85 (0.47)	6.38 (0.75)	6.05 (0.93)	6.17 (0.84)	< 0.001
School area						
Deprived	490 (59.3)	169 (71.6)	0 (0)	43 (45.3)	278 (77.4)	< 0.001
Non-deprived	337 (40.7)	67 (28.4)	137 (100)	52 (54.7)	81 (22.6)	
Weight status						
Normal	658 (79.6)	186 (78.8)	115 (83.9)	76 (80.0)	281 (78.3)	0.593
Overweight	137 (16.6)	42 (17.8)	19 (13.9)	13 (13.7)	63 (17.5)	
Obese	32 (3.9)	8 (3.4)	3 (2.2)	6 (6.3)	15 (4.2)	

^a^ Data were compared by the chi-squared test

At inclusion, 137 children met the criteria for overweight (16.6%) and 32 for obesity (3.9%; Table 3). Four years later, 101 children met the criteria for overweight (12.2%) and 31 for obesity (3.7%; *p* = 0.002). In children who remained overweight (*n* = 58), the BMI z-score decreased from 2.24 (0.48) to 2.04 (0.69; *p* = 0.014), but it remained unchanged in children with obesity [4.51 (1.82) vs. 4.09 (1.49)]. Weight status improved in half of the children with overweight/obesity (48.2 and 59.4%, respectively), with lower estimates in City 1 (38%) and City 2 (45.5%) and higher estimates in City 3 (68.4%) and City 4 (55.1%). Concomitantly, weight status worsened in 6.6% of children with overweight/normal weight.

**Table 3:** Weight status of schoolchildren at the first (2011) and last year (2015) of primary education according to weight status at inclusion (*N* = 827)

	First year	Change in weight status between the first and last year of primary education ^a^			Last year	
	N (%)	None	Negative	Positive	N (%)	*p*-value^b^
Normal	658 (79.6)	624 (94.8)	34 (5.2)	0 (0)	695 (84.0)	0.002
Overweight	137 (16.6)	58 (42.3)	13 (9.5)	66 (48.2)	101 (12.2)	
Obesity	32 (3.9)	13 (40.6)	0 (0)	19 (59.4)	31 (3.7)	

^a^ Coded “positive” if obesity changed to overweight/normal or if overweight changed to normal; “negative” if normal changed to overweight/obesity or if overweight changed to obesity

^b^: Data were compared by the McNemar test

The local implementers involved in training sessions were mainly municipal staff in charge of serving midday school meals, which is compulsory in France, and those in charge of designing and facilitating creative, interactive activities at school between and after classes. Over the 4-year study period, City 1 requested two training sessions on HE for the school catering staff, whereas City 2, City 3, and City 4 requested 3, 5, and 15 training sessions, respectively, on HE and PA for staff in charge of the school catering and staff in charge of ECAs. Finally, each participating municipality implemented a specific variation of the VIF program over the 4-year period (Table 4).

**Table 4:** Number (N) and child-staff ratios (CSRs) of school staff trained by occupation and training sessions attended in each participating municipality (*N* = 4) between 2011 and 2015

Training session	Healthy eating: HE						Physical activity		
School staff	Catering			ECA			ECA		
	N	CSR		N	CSR		N	CSR	
City 1	40	6:1	M						
City 2	20	7:1	M	12	12:1	M	25	6:1	M
City 3	20	5:1	L	30	4:1	L	30	4:1	L
City 4	127	3:1	L	79	5:1	L	63	6:1	M

ECA = facilitators of extracurricular activities

CSRs are expressed as number of children per adult, classified as low (L) if between 1 and 5 and as moderate (M) otherwise

In the univariate analysis (Table 5), estimated positive 4-year weight changes increased significantly with low CSRs compared to moderate CSRs, even after adjusting for sex, age, and deprived school area. When the four variations of the VIF program were entered as categorical covariates in the regression model (Table 6), the estimated positive 4-year weight change was significantly higher in low-to-moderate CSR multicomponent interventions than the reference (moderate CSR single-component intervention), and increased with age after adjustment. The moderate CSR multicomponent intervention had a similar effect as the reference program. In children with overweight/normal weight at inclusion (*N* = 795), the estimated 4-year negative weight change decreased with age and was unrelated to the process indicators being studied.

**Table 5:** Analysis of positive 4-year change in weight status according to child-staff ratios (CSRs) among children with overweight/obesity at inclusion (*N* = 169)

Staff: training	CSRs^a^	Children	Model 1	Model 2	Model 3
		N (%)	OR [95% CI]	OR [95% CI]	OR [95% CI]
School catering: HE	Low	97 (57.4)	**2.02 [1.09–4.48]**	**2.91 [1.44–5.87]**	**3.49 [1.67–7.29]**
	moderate	72 (42.6)	1	1	1
ECA facilitators: HE	Low	97 (57.4)	**2.23 [1.11–4.48]**	**3.51 [1.58–7.81]**	**3.39 [1.50–7.64]**
	Moderate	22 (13.0)	1.36 [0.49–3.75]	1.70 [0.60–4.90]	0.90 [0.27–3.02]
	None	50 (29.6)	1	1	1
ECA facilitators: PA	Low	19 (11.2)	**3.53 [1.15–10.9]**	**5.08 [1.58–16.4]**	**4.56 [1.38–15.05]**
	Moderate	100 (59.2)	1.84 [0.392–3.68]	**2.64 [1.22–5.71]**	**2.45 [1.12–5.38]**
	None	50 (29.6)	1		1

Significant results are bolded. Model 1: unadjusted estimates; Model 2: estimates adjusted for sex and age; Model 3: estimates adjusted for sex, age, and schooling in a deprived area. HE = healthy eating; PA = physical activity; ECA = extracurricular activity; OR = odds ratio; CI = confidence interval

^a^: Classified as low if between 1 and 5 children per adult, and as moderate otherwise

**Table 6:** Analysis of positive 4-year change in weight status among children with overweight/obesity at inclusion (*N* = 169) according to variations of the VIF program locally implemented in each participating municipality (Model 1), and adjusted for sex, age, and schooling in a deprived area (Model 2)

Variables				Children	Model 1	Model 2
				N (%)	OR [95% CI]	OR [95% CI]
Child Staff Ratios ^b^						
Variation^a^	School catering	ECA: HE	ECA: PA			
V4	*Low*	*Low*	*Low*	19 (11.2)	**3.53 [1.15–10.9]**	**4.32 [1.28–14.5]**
V3	*Low*	*Low*	*Moderate*	78 (46.2)	2.00 [0.97–4.14]	**3.18 [1.37–7.38]**
V2	*Moderate*	*Moderate*	*Moderate*	22 (13.0)	1.36 [0.49–3.75]	0.93 [0.28–3.12]
V1	*Moderate*	*None*	*None*	50 (29.6)		1
Characteristics at inclusion						
	Sex		Girls	93 (55.0)		0.78 [0.46–1.67]
			Boys	76 (45.0)		1
	Schooled in deprived area		Yes	107 (63.3)		0.45 [0.20–1.02]
			No	67 (36.7)		1
	Mean age					**1.65 [1.04–2.60]**

Significant results are bolded. HE = healthy eating; PA = physical activity; ECA = extracurricular activity; OR = odds ratio; CI = confidence interval

^a^ Numbered according to the city in which they were locally implemented

^b^ Classified as low if between 1 and 5 children per adult, and moderate otherwise. Based on occupation (school catering/facilitators of extracurricular activities) and training session attended (physical activity/healthy eating)

## Discussion

Between the first and the last year of primary education, weight status improved in half of the schoolchildren characterized as overweight/obesity at inclusion. In children who remained overweight, the BMI z-score diminished significantly over time, and being schooled in a deprived area had a negative, but not significant, influence.

The implementation mode under study was similar to traditional school-based prevention programs in that it provided training and materials to local stakeholders [12]. However, the VIF program provides tools previously tested in real-life settings and addresses thematic content over a long period of time based on a comprehensive social marketing methodology [30]. In addition, the interventions avoided interference with teachers’ curricula, enriching the existing school environments/interactions rather than implementing unusual and potentially disruptive procedures [13], and promoting experiential learning instead of lectures [19]. On the other hand, upstream discussions revealed that teachers had no particular interest in adding HE/PA to their curriculum, despite the recommendations of health experts [31]. Imposing their participation may have jeopardized local dynamics [13].

Yet, the data analyses elicited interesting insights. First, weight trajectories were reversed in half of children with overweight/obesity over 4 years, compared to approximately 38% over a 9-year period at the national level [2, 32]. Negative weight changes were marginal, and overweight severity diminished over time. This is important because 16.6% of children were considered overweight and 3.9% obese in the participating municipalities, compared to 10.1 and 2.4% of French children aged 6–10 years [2]. The higher than national average estimates could be due to the high level of social deprivation in these locations [33, 34], except for City 2*,* where others factors could be at play [35]. Second, positive changes in weight status were steadily related to lower CSRs, even after adjusting for sex, age, and deprived school area. Multi-component programs are widely acknowledged to be more successful than single-component interventions [36]. However, moderate CSRs weakened this benefit of the intervention in City 2, and the presence of low CSRs characterized the most effective variations in the VIF program. Many studies have investigated the relationship between CSRs and outcomes in childhood education and care, mostly on the cognitive and emotional development of the child, but the first attempt to systematically review and meta-analyze this highly complex and heterogeneous literature revealed few, if any, relationships [21]. These process indicators are often overlooked in obesity prevention program evaluations [10], and the reasons for variation at the local level warrant further examination. However, CSRs seem pivotal in childhood obesity prevention programs, though their optimal values still remain to be determined. Finally, it seems that older age had a positive influence on 4-year weight changes, though the children were relatively close to one another in regards to age (mean 6.38 years, SD 0.76). Minor differences in this life period could mark the transition between two milestones of cognitive development, but the complexity of developmental theories warrants caution [37]. If confirmed elsewhere, this result would advocate, at the very least, for including 2nd grade children in these programs.

The present study has limitations related, in part, to the implementation mode under examination. The avoidance of prescriptive approaches may have contributed to fostering local dynamics, but also precluded comparisons between balanced interventions. The lack of a control group constitutes a major limitation, as many factors besides the intervention could have influenced findings during the 4-year period. However, quasi experimental designs are frequently used to examine the effects of social programs [38], and assessing the intervention effect between the first and last year of primary education is consistent with monitoring school-based prevention programs. Furthermore, interactions between parents, children, and municipal school staff in charge of PA and HE were targeted by the programs but not directly assessed in the study. In addition, the municipalities participating in the study may have differed from other cities in France, as they systemically monitor the weight status of schoolchildren under their supervision. Considering the present findings, low CSRs in childhood prevention programs could be even more important in deprived areas [39]. This proportionate universalism [40] warrants further examination in relation to parental involvement and other variables of interest [35]. Nevertheless, a strength of this prospective study is that it relied on comparisons of reliable estimates of weight status collected twice at a 4-year interval among the same children at primary schools from distant municipalities, which avoided contamination.

## Conclusions

Our findings suggest that training ancillary school staff in experiential-focused interventions in locally implemented school programs contributed positively to reducing childhood obesity during the four years of primary education without interfering in educational activities. They also provide preliminary evidence that low CSRs could be pivotal for optimal outcomes, especially in deprived areas, which warrants further investigation using a controlled study design.

## Data Availability

The dataset generated and analyzed during the current study is available in the Open Science Frame Network repository at https://osf.io/bfd8r

## Abbreviations

VIF: Vivons en Forme (French: Live Healthy)
HE: Healthy Eating
PA: Physical activity
BMI: Body Mass Index
ECA: Extracurricular Activity
CSR: Chid-Staff Ratio
ZEP: (Zone d’éducation prioritaire: deprived area)
